# A case of cardiogenic shock due to acute coronary syndrome successfully recovered by percutaneous and paracorporeal left ventricular assist device

**DOI:** 10.1007/s10047-019-01101-x

**Published:** 2019-04-01

**Authors:** Makiko Nakamura, Masakazu Hori, Masaki Nakagaito, Hiroyuki Kuwahara, Osamu Kinoshita, Minoru Ono, Shigeki Yokoyama, Toshio Doi, Kazuaki Fukahara, Koichiro Kinugawa

**Affiliations:** 1 The Second Department of Internal Medicine, University of Toyama, 2630 Sugitani, Toyama-City, 930-0194 Japan; 2 Department of Cardiac Surgery, University of Tokyo, Tokyo, Japan; 3 Department of Cardiovascular Surgery, University of Toyama, Toyama, Japan

**Keywords:** Impella, Ventricular assist device, Left main trunk, Cardiogenic shock, Bridge to recovery

## Abstract

We recently experienced a 70-year-old woman with left main trunk-acute coronary syndrome who was initially supported by Impella 5.0 which converted to paracorporeal left ventricular assist device (LVAD) implantation as a bridge to recovery. Optimized guideline-directed medical therapy with cardiac rehabilitation resulted in successful explantation of LVAD and she discharged on foot.

## Introduction

The prognosis of acute myocardial infarction (AMI) patients with cardiogenic shock has been still poor in use of intra-aortic balloon pump (IABP) or veno-arterial extracorporeal membrane oxygenation (VA-ECMO) [1–4]. Impella provides superior hemodynamic support that is characterized as marked reduction in left ventricular (LV) preload [5]. Theoretically, mechanical unloading may facilitate myocardial recovery [6]. Here, we present a case of cardiogenic shock due to left main trunk (LMT)-AMI and severe mitral regurgitation (MR). Our initial strategy for LV unloading by Impella and paracorporeal LV assist device (LVAD) resulted in the recovery of cardiac function and successful freedom from mechanical circulatory support (MCS).

## Case report

The patient was a 70-year-old woman who was hospitalized to a secondary care hospital for chest pain. Electrocardiogram (EKG) showed atrial fibrillation (AF) rhythm and ST elevation in I, aVL, aVR, and V1-3 leads. Transthoracic echocardiography (TTE) showed moderate MR. Coronary angiography (CAG) revealed 90% stenosis in LMT. Her chest pain and ST elevation spontaneously resolved during CAG, and elective bypass surgery was scheduled. In the next early morning, she developed AMI by LMT occlusion, and fell into cardiogenic shock. Emergent PCI with IABP support was performed, and drug eluting stents were placed in the LMT with TIMI 3 flow. Peak CPK level was 2696 IU/L at 12 h after AMI onset and IABP was weaned off on the next day. But her hemodynamics was unstable despite incremental dose of intravenous inotropes, and she was referred to us for further intensive care 2 days after AMI onset.

**Fig. 1:**
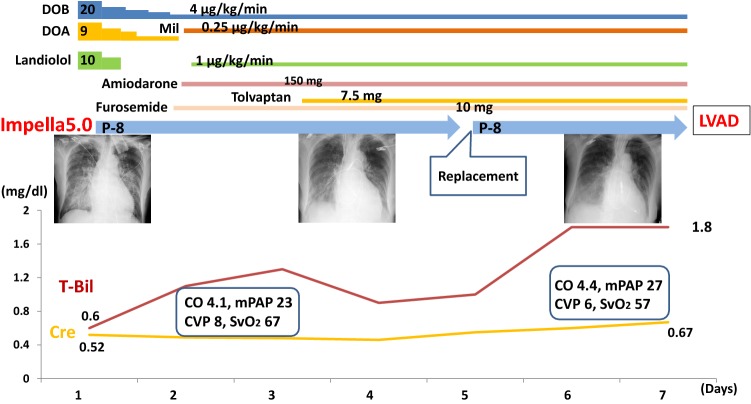
The clinical course after Impella 5.0 insertion until LVAD implantation. The dose of administered drugs was also shown. *DOB* dobutamine, *DOA* dopamine, *Mil* milrinone, *T-Bil* total bilirubin, *Cre* serum creatinine *(L)VAD* (left) ventricular assist device, *mPAP* mean pulmonary artery pressure, *SvO*_*2*_ mixed venous oxygen saturation, *CO* cardiac output, *CVP* central venous pressure

On admission to our hospital, chest X-ray showed severe pulmonary congestion regardless of high doses of inotropes (Fig. 1). TTE showed reduced LV ejection fraction of 32% with severe MR and mild aortic regurgitation (AR). Laboratory test results were unremarkable except markedly elevated B-type natriuretic peptide (BNP) level. Lactate level was 1.3 mmol/L.

For the initial MCS, we inserted Impella 5.0 from her right femoral artery immediately after hospitalization, i.e., 2 days after AMI onset. Approximately, 4.3 L/min of pump flow was obtained at P-8 level. We could reduce the dose of intravenous inotropes and lung congestion gradually improved, but AF tachycardia relapsed and MR remained severe. After 1 week, high pulmonary artery pressure and low mixed venous oxygen saturation as well as an increase in total bilirubin level taught us a difficulty to wean from Impella (Fig. 1). Considering relatively low-peak CPK level, we discussed about a future possibility of weaning from MCS if we could control MR and AF tachycardia. On the 7th day, we replaced mitral valve (MVR), grafted saphenous vein to LAD, isolated pulmonary vein, resected left atrial appendage, and implanted paracorporeal Nipro-VAD as a bridge to recovery. Intraoperative findings revealed that a chordae tendineae of the anterior leaflet (A2) of the mitral valve was ruptured.

**Fig. 2:**
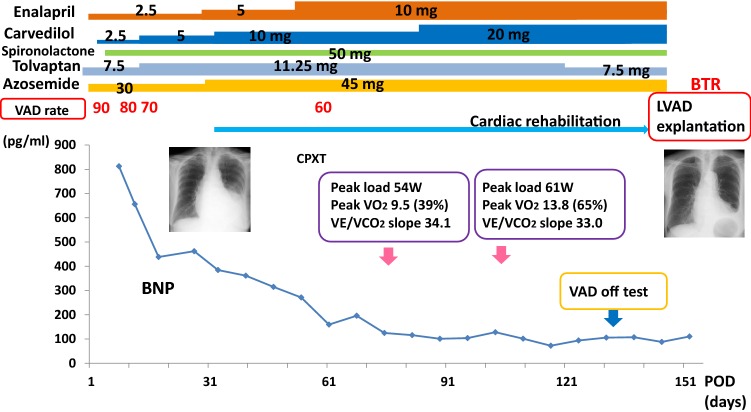
The clinical course after LVAD implantation. *(L)VAD* (left) ventricular assist device, *BNP* B-type natriuretic peptide, *CPXT* cardiopulmonary exercise testing; *POD* post-operative day

Two weeks after operation, TTE showed aortic valve opening on every heart beat with mild to moderate AR. We started and titrated enalapril and carvedilol (Fig. 2). We reduced the support of LVAD over 2 months, and at the same time started cardiac rehabilitation. On the 79th POD, the 1st cardiopulmonary exercise (CPX) testing revealed that peak work load, peak oxygen consumption (peak VO_2_), and VE/VCO_2_ slope were 54 W, 9.5 mL/kg/min (39% of normal), and 34.1, respectively. The 1st “off-test” revealed that pulmonary artery wedge pressure (PAWP) was elevated during LVAD was stopped (Table 1), and we considered that her heart function was not ready to be free from MCS. We further titrated carvedilol to 20 mg daily and strengthened rehabilitation. On the 111th POD, the 2nd CPX testing showed that the peak work load, peak VO_2_, and VE/VCO_2_ slope were improved to 61 W, 11.8 mL/kg/min (65% of normal), and 33.0, respectively. The 2nd off-test revealed that PAWP during LVAD-off again elevated and cardiac output (CO) was not increased by saline loading.

**Table 1:** Hemodynamic results of VAD-off test

	1st (POD 83)	2nd (POD 120)	3rd (POD 146)
RAP at baseline (mmHg)	3	5	5
PAWP at baseline (mmHg)	12	11	13
PAP at baseline (mmHg)	29/12 (19)	26/14 (18)	32/10 (20)
CO/CI at baseline (L/min)/(L/min/m^2^)	2.94/2.13	3.69/2.31	2.81/2.10
PAWP during VAD-off (mmHg)	20	20	16
CO/CI during VAD-off (L/min)/(L/min/m^2^)	2.89/2.10	2.91/2.17	2.47/1.85
PAWP after saline infusion (mmHg)		26	13^a^
CO/CI after saline infusion (L/min)/L/min/m^2^)		2.92/2.18	3.34/2.49^a^

*POD* post-operative day, *RAP* right atrial pressure, *PAWP* pulmonary artery wedge pressure, *PAP* pulmonary artery pressure, *CO* cardiac output, *CI* cardiac index, *VAD* ventricular assist device

^a^Under sedation

We then considered if the cause of PAWP elevation might be attributable to AR, since AR appeared to be moderate at TTE. Limited visible range of TTE, partly because of previous open heart surgery, made it difficult for accurate evaluation of AR grade, and we performed aortography that showed third degree of AR by the Sellers classification. The 3rd off-test revealed that saline loading resulted in a significant increase in CO without elevation of PAWP (Table). On the 155th POD, we successfully explanted LVAD after AVR on the same day. According to the operative findings, aortic valve had three cusps with mild fusion of each commissure and non-coronary cusp was relatively small and moderately thickened. She was discharged from our hospital on foot with LVEF of 35% and BNP of 210 pg/mL. She was alive and well as of 4 months after discharge.

## Discussion

In this report, we described a case of 70-year-old ischemic cardiomyopathy who received MVR and paracorporeal LVAD implantation after 1-week support of Impella. All MCSs were successfully withdrawn after 5 months with significant recovery of cardiac function.

On admission, this patient had severe lung congestion. Impella has been shown to increase CO with lowered PAWP [5]. VA-ECMO, which generally increases LV afterload, might have got pulmonary congestion worse if it had been applied. We could not implant paracorporeal LVAD at this early stage because of her age ineligible for transplant listing. Therefore, we considered that Impella was the best selection for the first MCS.

Her hemodynamics was stabilized by Impella, but we could not wean it by severe MR and recurrent AF. If destination therapy was approved, the choice for the 2nd MCS might be implantable LVAD, but our strategy now should be best supportive care or paracorporeal LVAD implantation aiming for bridge to recovery.

Mechanical unloading by LVAD with guideline-directed medical therapy (GDMT) sometimes leads to LV reverse remodeling (LVRR), or even makes possible to remove LVAD [7–9]. We previously reported that patients with shorter history of heart failure had a better chance to achieve LVRR under LVAD support [10], but it rarely happened in patients with ischemic etiology. Predictors of recovery have been reported: age < 50 years, non-ischemic etiology, history of cardiac diseases < 2 years, etc. [6]. Considering older age and ischemic etiology of this case, odds were against LVRR. On the other hand, lower 24-h CK-MB was reported to associate with lower MACE at 2 years’ follow-up among patients who received primary PCI for anterior STEMI [11]. She never had a history of heart failure and the peak CPK level was relatively low. Pulsatile flow may have advantages to induce LVRR over continuous flow [12–14]. Therefore, we considered that she had a good chance for LVRR during pulsatile LVAD support if we simultaneously managed complications.

We also previously reported a scoring system using the values of peak load, peak VO_2_, and VE/VCO_2_ slope, to predict future explantation of LVAD [14]. Her score was 1 point and the probability of LVAD withdrawal was 29% at the 1st CPX testing. Her score increased to 3 points and the probability of LVAD withdrawal was 86% at the time of the 2nd CPX testing. The usefulness of LVAD-off test with saline infusion has been reported [15, 16]. In our case, there was no elevation of PAWP but a significant increase in CO after saline infusion. Collectively, we considered that her cardiac function recovered enough to tolerate LVAD explantation.

In this case, chordal rupture with flail anterior (A2) scallop of the mitral valve was observed at the MVR and Nipro-VAD implantation. A case of acute MR due to chordal rupture and flail mitral valve leaflet was reported during Impella replacement [17]. Impella could also cause an iatrogenic injury of the aortic valve [18]. In this case, already existing mild AR in the native valve might be worsened to moderate by the insertion of Impella or blood supply by Nipro-VAD into ascending aorta, which eventually required the valve replacement at the time of VAD explantation.

In summary, a 70-year-old patient with refractory heart failure of ischemic etiology complicated with combined valvular disorders was initially treated by MCS and successfully bridged to recovery. We cannot emphasize too much the importance of early ventricular unloading that may facilitate myocardial recovery. Additionally, we should underscore aggressive combination therapy including titration of GDMT and cardiac rehabilitation with respect to bring reverse remodeling.
